# Advances and New Concepts in Alcohol-Induced Organelle Stress, Unfolded Protein Responses and Organ Damage

**DOI:** 10.3390/biom5021099

**Published:** 2015-06-03

**Authors:** Cheng Ji

**Affiliations:** GI/Liver Division, Research Center for Liver Disease, Department of Medicine, Keck School of Medicine of USC, University of Southern California, Los Angeles, CA 90033, USA; E-Mail: chengji@usc.edu; Tel.: +1-323-442-3452

**Keywords:** alcohol, UPR, proteostasis stress responses, inter-organellar crosstalk, organ injuries

## Abstract

Alcohol is a simple and consumable biomolecule yet its excessive consumption disturbs numerous biological pathways damaging nearly all organs of the human body. One of the essential biological processes affected by the harmful effects of alcohol is proteostasis, which regulates the balance between biogenesis and turnover of proteins within and outside the cell. A significant amount of published evidence indicates that alcohol and its metabolites directly or indirectly interfere with protein homeostasis in the endoplasmic reticulum (ER) causing an accumulation of unfolded or misfolded proteins, which triggers the unfolded protein response (UPR) leading to either restoration of homeostasis or cell death, inflammation and other pathologies under severe and chronic alcohol conditions. The UPR senses the abnormal protein accumulation and activates transcription factors that regulate nuclear transcription of genes related to ER function. Similarly, this kind of protein stress response can occur in other cellular organelles, which is an evolving field of interest. Here, I review recent advances in the alcohol-induced ER stress response as well as discuss new concepts on alcohol-induced mitochondrial, Golgi and lysosomal stress responses and injuries.

## 1. Proteostasis and Alcohol Metabolites: An Overview

Proteins are essential and large biomolecules that participate in virtually every process within the cell and affect a vast array of functions of living organisms. To ensure efficient functioning, a homeostasis or an optimal state of the entire set of proteins (the proteome) expressed by a genome at a given time and under defined conditions must be maintained [1]. The protein homeostasis or proteostasis balances the biogenesis, folding, trafficking and degradation of proteins within and outside the cell, which is of central importance to the health of the cell and lifespan of organisms. A few crucial regulations are required to maintain the proteostasis and dynamic composition of the proteome. The first regulation occurs during protein translation, in which the information required for the folding of polypeptide chains into functional three-dimensional native conformations is encoded in the primary sequence and shaped by the structure of the ribosome [2]. The second regulation is post-translational. Molecular chaperones and/or chaperonins help in the assembly, disassembly, folding and stability of proteins in the crowded cellular environment. The third regulation is through molecular machines for protein degradation. Damaged or abnormal proteins are selectively degraded by the ubiquitin-proteasome system (UPS). Unfolded and misfolded proteins in the endoplasmic reticulum (ER) are targeted and eliminated through a process called ER-associated degradation (ERAD) that involves the UPS. ERAD is activated by the unfolded protein response (UPR) which senses ER stress, a condition under which unfolded or misfolded proteins accumulate [3]. Unwanted proteins can also be removed by autophagy, an intracellular degradation system that delivers cytoplasmic constituents to the lysosome [4]. Collectively, these regulations and cellular mechanisms safeguard quality of proteins and maintain the proteostasis. A loss of the proteostasis activates various cellular stress responses leading to either adaptation or disease development. Typically, the UPR singling in the ER attenuates protein translation, increases expression of chaperones, and increases efficiency in protein trafficking and degradation, which help alleviate the effects of ER stress and restore the ER homeostasis [5]. However, severe or prolonged ER stress from constitutive expression of abnormal proteins or environmental conditions such as pathogen infection or drug abuse, triggers cell death pathways and inflammation, which could dramatically disturb the proteostasis resulting in a variety of diseases such as cystic fibrosis, Alzheimer’s disease, diabetes, obesity, and alcohol-related pathologies [6].

Alcohol is a simple and consumable biomolecule which crosses the biological membrane and is readily distributed throughout the body. Excessive consumption of alcohol disturbs numerous biological pathways causing damage to many organs such as the liver, brain, pancreas, heart, bones, and immune system [7,8,9,10,11,12]. In the body, alcohol is oxidized by cytosolic alcohol dehydrogenase (ADH) to acetaldehyde, which is oxidized by aldehyde dehydrogenase (ALDH) into acetate entering the circulation. Cytochrome p450 activities, mainly CYP2E1 in the ER of liver cells also oxidize alcohol to acetaldehyde and hydrogen peroxide (H_2_O_2_). Alcohol and its derivatives are highly reactive and can modify proteins directly [13,14,15,16,17]. At physiological temperature and pH, acetaldehyde reacts with nucleophilic groups in proteins, such as α-amino groups of internal lysine residues and the ε-amino group on the N-terminal amino acid of unblocked proteins, which forms unstable Schiff base acetaldehyde adducts. Alcohol consumption is also known to elevate blood homocysteine (Hcy), which is a normal intermediate in the metabolism of the essential amino acid methionine. Homocysteine thiolactone (HTL) derived from Hcy during protein synthesis undergoes not only nucleophilic reactions, which can be facilitated in the presence of acetaldehyde, but also electrophilic reactions to form protein adducts or homocysteinylated proteins, causing malfolding and triggering the ER stress response [18]. Chronic ethanol consumption increases the production of superoxide, H_2_O_2_, lipid peroxides, or peroxynitrite [19,20,21], which breaks the redox status of the ER and perturbs the oxidative protein folding. These biological and metabolic features link alcohol consumption to interference of the proteostasis, the UPR and subsequent pathologies, which may occur not only in the ER but also in other organelles such as the Golgi apparatus, mitochondria and lysosomes [22,23,24]. This review aims to highlight advances in alcohol-induced ER stress response and discuss potential molecular effects of alcohol on other organelle protein stress responses.

## 2. Alcohol and UPR in the ER

**Figure 1:**
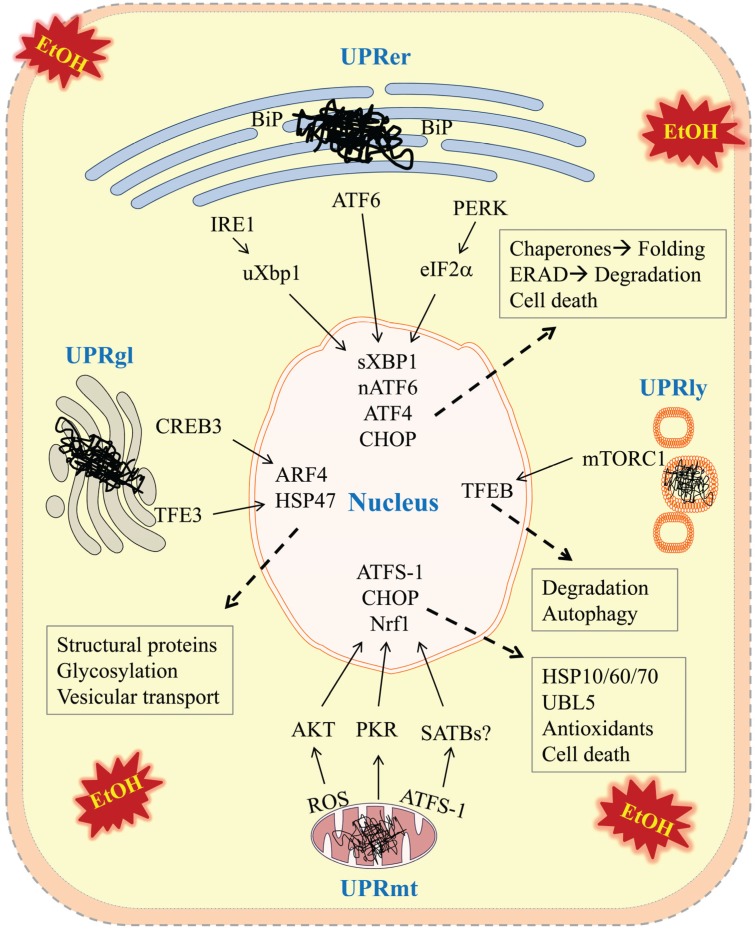
Potential Molecular Mechanisms of Alcohol-induced Unfolded Protein Responses in Endoplasmic Reticulum, Golgi Apparatus, Mitochondria, and Lysosomes. Alcohol and/or its metabolites induce accumulations of unfolded/misfolded proteins (depicted by 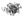) in the four types of cellular organelles, which create a stress condition in each of the organelles. The stress activates specialized transcriptional programs (solid arrows) mediated by distinct transducer(s) in each organelle leading to either restoration of organellar protein homeostasis or contribution to cell death (dashed arrows). EtOH, alcohol and/or alcohol metabolites; UPRer or UPR, unfolded protein response in the endoplasmic reticulum (ER); BiP/GRP78, binding immunoglobulin protein also known as 78 kDa glucose-regulated protein; IRE1α, inositol requiring enzyme 1α; ATF 4 or 6, activating transcription factor 4 or 6; nATF6, nuclear ATF6; PERK, PKR-like ER-localized eIF2α kinase; eIF2α, eukaryotic translation initiation factor 2α; uXBP1, un-spliced X-box binding protein 1; sXBP1, spliced XBP1; ERAD, ER associated degradation; CHOP, C/EBP homology protein 10. UPRgl, unfolded protein response in the Golgi apparatus; CREB3; a basic leucine zipper-containing transcription factor; TFE3, a basic-helix-loop-helix type transcription factor; ARF4, a member of the small GTPase family that regulates Golgi-to-ER vesicular transport; HSP47, a 47 kDa collagen-binding glycoprotein. UPRmt, unfolded protein response in the mitochondria; ROS, reactive oxygen species; ATFS-1, activating transcription factor associated with stress-1; AKT, also known as protein kinase B (PKB), is a serine/threonine-specific protein kinase; PKR, protein kinase RNA-activated also known as protein kinase R; Nrf1, the nuclear factor erythroid 2-related factor 1; SATBs, special AT-rich sequence-binding proteins; UBL5, ubiquitin-like 5; HSP10, 60 or 70, heat shock protein 10, 60 or 70 acting as mitochondrial chaperones. UPRly, unfolded protein response in the lysosomes; mTORC1, mammalian target of rapamycin complex 1; TFEB, transcription factor EB.

Alcohol induced ER stress response was initially reported in the liver of alcohol-fed mice, which involves ER chaperones (e.g., BiP/GRP78, binding immunoglobulin protein also known as 78 kDa glucose-regulated protein), three ER resident sensors (*i.e.*, IRE1 (inositol requiring enzyme 1), ATF6 (activating transcription factor 6), and PERK (double-stranded RNA-activated protein kinase (PKR)-like ER kinase), and transcription factors such as Xbp1 (X-box binding protein 1) and CHOP (C/EBP homology protein 10) (Figure 1) [25]. This initial discovery drew immediate attention as alcohol is mainly metabolized in the ER-rich hepatocytes. It is now known that alcohol-induced ER stress response occurs not only in liver but also in other major organs such as brain, pancreas and heart, and not only in rodents but also in other species, as well as in human alcoholics [23,24,25,26,27,28,29,30,31]. Several lines of molecular evidence substantially support an important role of alcohol-induced ER stress in damage of various organs, and disease development.

In the liver of mice fed alcohol, selected ER stress markers are associated with severe steatosis, scattered apoptosis and necroinflammatory foci [25]. In micropigs fed alcohol, liver steatosis and apoptosis were shown to be accompanied by increased mRNA levels of CYP2E1 and GRP78 and protein levels of CYP2E1, GRP78, activated SREBP and caspase 12 [28,29]. The ER stress response correlated with elevated transcripts of lipogenic enzymes such as fatty acid synthase (FAS) and acetyl-CoA carboxylase (ACC). In baboons fed alcohol orally, increased calpain 2, calpain p94, and ERD21 and decreased eIF2α were among the genes with altered expression by large-scale cDNA array analyses [30]. Alcohol and lipopolysaccharide (LPS) were shown to impair UPR during liver cirrhosis [31]. LPS activated UPR markers such as IRE1α, ATF-6 and eIF2α (eukaryotic translation initiation factor 2A), but sensitized cirrhotic livers to LPS/TNFα-mediated apoptosis. Further, gene expression profiling of cirrhotic liver samples from human alcoholics also revealed alterations of calpain and calreticulin that are indicative of ER malfunction [30].

Similar to that of the liver, the pancreas has a high rate of protein synthesis and turnover and the capacity to metabolize alcohol yielding toxic metabolites such as acetaldehyde and lipid esters. Acute pancreatitis is often associated with perturbations of ER homeostasis [32,33,34], in which all three ER stress/UPR transducers and their downstream pathways are activated. However, alcohol feeding activated the UPR and XBP1 without causing an apparent injury in pancreatic tissues [33,34]. This suggests that alcohol induces physiologic adaptive UPR preventing pathophysiologic consequences in the pancreas. Indeed, heterozygous deletion of the XBP1 gene prevents XBP1 upregulation and results in pathologic changes including extensive dilation of the ER with occasional dense luminal inclusions as well as significant accumulation of autophagic vacuoles in acinar cells [33,34]. Thus, alcohol-induced UPR is protective in the pancreas and additional risk factors may be required for pancreatic damage to occur.

In the heart, ER stress plays a critical role in regulating protein synthesis in cardiac myocytes, and thereby produces cell enlargement and cardiac hypertrophy. Chronic (>7 days) alcohol consumption by Friend virus-B type (FVB) albino mice resulted in increased heart weight and heart-to-body weight ratio [35]. Protein expression levels of GRP78, CHOP, and IRE1α were increased. Overexpressing alcohol dehydrogenase in the FVB mice during alcohol exposure resulted in an enhanced ER stress. Furthermore, LPS-induced maximal shortening velocity of cardiac myocytes was inhibited upon overexpression of the antioxidant protein metallothionein [36], which was correlated with increased protein levels of GRP78, CHOP, PERK and IRE1α and decreased PERK, phospho-JNK and phospho-p38 [35,36].

In the brain, alcohol exposure is known to cause loss of neurons in the developing central nervous system (CNS). Alcohol consumption during pregnancy causes fetal alcohol spectrum disorders (FASDs) [8,21]. Children with FASD have a variety of cognitive, behavioral and neurological impairments [21]. Given that ER stress plays a significant role in the pathogenesis of several popular neurological diseases such as brain ischemia and Alzheimer’s disease [37,38,39,40], ER stress may also be involved in alcohol-induced neuron toxicity. In fact, exposure of primary cerebellar granule neurons to alcohol markedly increased the expression of GRP78, CHOP, ATF4, ATF6, and phosphorylated PERK and eIF2α in the presence of an ER stress-inducing agent, tunicamycin or thapsigargin, which was accompanied with increased cell death [41,42]. In infant mice at an age equivalent to the third trimester of pregnancy in humans, acute alcohol exposure by subcutaneous injection significantly increased ATF6, CHOP, GRP78, mesencephalic astrocyte-derived neurotrophic factor, and phosphorylation of IRE1, eIF2α and PERK in neurons of the cerebral cortex, hippocampus and thalamus, which indicates that alcohol alone induces ER stress in the developing brain. In addition, in adult and aging rats following 10-month alcohol consumption, the ER stress and ER Ca^2+^ ATPase pump (SERCA) were found to be associated with structural and functional alterations of the dendritic arbor of Purkinje neurons [43].

With respect to molecular causes of alcohol-induced ER stress, there are new candidates being identified besides the aforementioned formation of protein adducts and disturbance of ER redox status that have been reviewed previously [23,24]. The new candidates include alcohol-induced epigenetic changes, iron overload, zinc deficiency, circadian clock, altered ER membrane lipid composition, and disrupted Ca^2+^ homeostasis. First in a study with cystathionine β synthase (CBS) heterozygous mice [44], the liver S-adenosylmethionine (SAM) to S-adenosylhomocysteine (SAH) ratio negatively correlated with alcohol-induced steatohepatitis. In parallel, the SAM to SAH ratio correlated to reduced levels of 3meH3K9 (the suppressor chromatin marker trimethylated histone H3 lysine-9) in the promoter regions of ER stress response markers [44]. In another study using mice with liver specific knockout of the Grp78 [45], long-term alcohol feeding exerted strong effects on the methylation of CpG islands at promoter regions of the ERAD genes including derlin 3, Creld2 (cysteine-rich with EGF-like domains 2), Herpud1 (ubiquitin-like domain member), Wfs1 (wolfram syndrome gene), and Yod1 (deubiquinating enzyme 1). These epigenetic methylation changes were associated with severe liver tumorigenesis in the alcohol-fed mice, indicating an epigenetic cause in alcohol-induced ER stress and injury. Second, evidence for a causal effect of iron overload on alcohol-induced ER stress was reported in mice deficient in the hemochromatosis gene (Hfe^−/−^). Co-feeding *ad libitum* with alcohol and a high-fat diet (HFD) was found to lead to increased XBP1 splicing, activation of IRE-1α and PERK, and CHOP protein expression, which were associated with profound steatohepatitis and fibrosis [46]. Third, in contrast to iron overload, alcohol induces zinc deficiency in alcoholics, which links to ER stress and cell death damage. In Wistar rats fed chronic alcohol, it was found recently that increased expression of hepatic p-eIF2α, ATF4 and CHOP and activation of caspase-3 were associated with increased cell death and reduced hepatic and hepatocyte ER zinc levels. The alcohol-induced ER stress and cell death could be inhibited by zinc supplement but not by antioxidant treatment [47]. Fourth, the circadian clock has recently been found to be associated with alcoholic ER stress and fatty liver injury [48]. Alcohol perturbs the nuclear receptor SHP (small heterodimer partner)-mediated circadian clock and impairs oscillations of the ER stress response and lipid metabolism resulting in fat accumulation. Lastly, alterations of lipid composition by alcohol can be a direct cause for ER stress. One of the common phospholipids of membrane bilayers is sphingomyelin. Acid sphingomyelinase (ASMase) hydrolyzes sphingomyelin into ceramides, which in addition to their critical structural function in membrane bilayers regulate apoptosis, cellular stress response, inflammation and metabolism [49,50,51,52]. Alcohol feeding increases ASMase expression and activity in experimental models and patients with acute alcoholic hepatitis [53]. ASMase is required for alcohol-induced ER stress [54]. Particularly, incubation of HepG2 cells (liver hepatocellular carcinoma cell lines) with exogenous ASMase disrupts ER Ca^2+^ homeostasis [53,54]. Since SERCA contributes to Ca^2+^ homeostasis in the ER, it is likely that ASMase activation and subsequent ceramide production disrupts physical properties of the ER membrane, which modulates SERCA activities [54]. Thus, SERCA activities might be a key element in alcohol-induced ER stress and injury. Other earlier experimental results also support this notion. In mice, alcohol exposure aggravates the inhibitory effects on SERCA and Ca^2+^ homeostasis by some anti-HIV drugs [55]. In a model for dendritic regression of purkinje neurons from the cerebellar cortex of ethanol-fed rats, dilation of the extensive smooth ER and altered SERCA activities were shown to precede activation of ER resident caspase 12 [43]. The above findings and advances shed new light on what causes alcohol-induced ER stress response and injuries.

## 3. Alcohol and UPR in the Mitochondria

Following the concept of UPR and protein homeostasis in the ER, researchers have been exploiting similar mechanism in the mitochondria. Mitochondria are complex organelles and essential for cell viability and ATP generation through oxidative phosphorylation. About 1000 proteins are present in the mitochondria. Most of them are nuclear encoded, translated in the cytoplasm and transported to the mitochondria. A small portion of mitochondrial proteins are encoded by the mitochondrial genome and synthesized and folded in the mitochondria [56]. Some mitochondrial encoded proteins form complexes with nuclear encoded proteins. Thus, the mitochondrial protein homeostasis may regulate not only synthesis and folding of proteins within the mitochondria but also synthesis, folding and import of proteins outside the mitochondria. Any stress condition that disturbs the balance between protein synthesis and turnover in the mitochondria can cause protein accumulation and aggregation, which presumably activate a mitochondrial unfolded protein response (referred to as UPRmt) to maintain mitochondrial protein homeostasis [57,58].

Relevant molecular evidence supporting the existence of UPRmt was initially from studies on genetically modified Caenorhabditis elegans [59,60]. In these studies, upon UPRmt, mitochondria specific chaperones HSP60 and HSP70 were found to be increased. Accumulated unfolded proteins in the mitochondria were degraded to peptides by an AAA-ATPase protease, CLPP1. The resultant peptides were transported to the intermembrane space of the mitochondria and released into the cytoplasm, where they inhibited transportation of transcription factors with specific targeting signals for mitochondrial transport [61]. ATFS-1 (activating transcription factor associated with stress-1) and DVE-1 were the two identified transcription factors. An inhibition of ATFS-1 and DVE-1 leads to activation of transcription of mitochondria-related genes such as mitochondrial chaperones such as HSP10, HSP60 and HSP70 and proteases such as UBL5 (ubiquitin-like 5) (Figure 1 and Table 1) [62]. Since mammalian homologs of DVE-1 are SATBs (special AT-rich sequence-binding proteins) that are the global chromatin organizers [63], DVE-1 together with ATFS-1 may increase the expression of mitochondrial chaperone genes through chromatin remodeling. However, such pathways have not been revealed in mammalian cells. Other pathways relevant to UPRmt appear present in mammals. For instance, in rat hepatoma p0 cells that are under UPRmt due to a loss of mitochondrial genome or expression of mutated ornithine transcarbamylase in the mitochondrial matrix, PKR (protein kinase RNA-activated also known as protein kinase R) phosphorylates eIF2α attenuating the protein translation. CHOP that was originally identified to regulate ER stress-related cell death has been found to regulate the expression of mitochondrial chaperones such as HSP10 and HSP60 and matrix proteases such as DnaJ and ClpP [64,65]. Upstream, CHOP is regulated by the AP-1 enhancer element and the MEK-JNK2-cJun pathway in response to the accumulation of unfolded proteins in the mitochondrial matrix [66]. Downstream, CHOP forms a heterodimer with C/EBPβ and activates the transcription of many mitochondrial genes include YME1L1 (ATP-dependent zinc metalloprotease), TIM17A (import component), NDUFB1 (Complex I subunit), endonuclease G and thioredoxin 2. The UPRmt can also be activated upon expression of a mutant form of endonuclease G, which caused overproduction of ROS, leading to activation of the kinase AKT in mammals [67]. AKT phosphorylates and activates the estrogen receptor α (ERα) localized on the mitochondrial membrane. ERα was shown to activate a transcription factor Nrf1 (the nuclear factor erythroid 2-related factor 1), which is responsible for the transcription of genes encoding antioxidant and mitochondrial respiratory chain components, protecting mitochondria from loss of mitochondrial protein homeostasis. ERα also increases the activity of the proteasome, and a mitochondrial protease OMI that degrades unfolded proteins in the mitochondria [68]. Thus, mechanisms of UPRmt in mammalian cells may be different from those in *C. elegans*.

**Table 1:** Potential factors involved in alcohol-induced organelle stress responses.

Organelle	Molecular Factors	References
Mitochondria	Activating transcription factor associated with stress (ATFS)	[23,24,56,57,58,59,60,61,62,63,64,65,66,67,68,69,70,71,72,73,74,75]
	Special AT-rich sequence-binding proteins (SATBs)	
	Protein kinase RNA-activated/protein kinase R (PKR)	
	Serine/threonine-specific protein kinase (AKT)	
	C/EBP homology protein 10 (CHOP)	
	Estrogen receptor a (ERa)	
	The nuclear factor erythroid 2-related factor 1 (Nrf1)	
	Ubiquitin-like 5 (UBL5)	
	The mitochondrial specific deacetylase (SIRT3)	
	Heat shock protein 10, 60 or 70 (HSP10, 60, or 70)	
Golgi	The basic-helix-loop-helix type transcription factor (TFE3)	[76,77,78,79,80,81,82,83,84,85,86,87,88,89,90,91,92,93,94,95,96,97,98,99,100,101,102,103,104,105]
	The basic leucine zipper-containing transcription factor (CREB3)	
	Member of the small GTPase family that regulates Golgi-to-ER vesicular transport (ARF4)	
	The 47 kDa collagen-binding glycoprotein (HSP47)	
	Glycosyl transferase	
Lysosomes	Mammalian target of rapamycin complex 1 (mTORC1)	[106,107,108,109,110,111,112,113,114,115,116,117,118,119,120,121,122,123,124,125,126,127]
	Transcription factor EB (TFEB)	
	Eukaryotic initiation factor 4E-binding protein 1 (4E1-EBP1)	
	Unc-51 like autophagy activating kinase 1 (ULK1)	
	Eukaryotic initiation factor 4G/4E (eIF4G/4E)	
	The Bcl-2e associated athanogen 3 (BAG3)	
	Ribosomal protein S6 kinase (S6K)	

Direct or indirect evidence is emerging for alcohol-induced disruption of mitochondrial protein homeostasis and UPRmt. In alveolar type 2 cells isolated from alcohol fed mice, the boldface mitochondrial proteins are significantly altered in alcohol *versus* caloric control-fed animals [69]. In the liver, chronic ethanol-mediated oxidative stress and lipid peroxidation increases the levels of various reactive species including 4-hydroxynonenal (4-HNE). 4-HNE can modify proteins in the liver [70]. In ethanol-fed rats, 4-HNE modified proteins were detected, which include electron transfer flavoprotein α (ETFα), dimethylglycine dehydrogenase, enoyl CoA hydratase and cytochrome C. Whether these modified proteins undergo unfolding or mis-folding in the mitochondria needs to be further investigated. However, in another study with mice deficient in the mitochondrial specific deacetylase Sirt3, proteins involved in the folding function such as calreticulin, HSP70, heat shock cognate 71 kDa protein, and hypoxia up-regulated protein 1 (HYOU1) were increased in response to alcohol feeding [71], suggesting a role of mitochondrial protein acetylation and modification in the UPRmt. Interestingly, some alcoholic UPRmt factors play a role in the mitochondria different from that in the ER. For instance, CHOP has long been known to be involved in alcohol-induced ER stress and cell death injury [23,24]. In mice deficient in CHOP, alcohol induced ER stress responses are detectable. However, alcohol-induced cell death is only partially reduced in the absence of the transcription of CHOP-regulated pro-apoptotic genes [72]. This suggests a possibility that mitochondria modulate alcohol-induced cell death through a protective role of CHOP during the UPRmt. In support of this notion, there is evidence showing that CHOP binding to an enhancer element of consensus sequence GGTTGCA activates transcription of mitochondrial chaperone and protease genes during the UPRmt [73]. In addition to the modulation of cell death, alcohol-induced UPRmt also contribute to liver tumorigenesis. In mice deficient in chaperone BiP and with constitutive ER stress, long-term (12 months) alcohol feeding induces liver tumor development. Overexpression of an isoform of ERα is significantly associated with the rate for liver tumor occurrence [74,75]. Since the aforementioned ERα is a downstream target of UPRmt, it is likely that impaired protein homeostasis in both mitochondria and the ER contributes to alcohol-induced tumorigenesis.

## 4. Alcohol and UPR in the Golgi

The Golgi apparatus is part of the cellular endomembrane system in which secretory and membrane proteins receive various modifications such as glycosylation and sulfation and are packaged into membrane-bound vesicles inside the cell before the vesicles are sent to their destination [76]. Like the ER, the structure and capacity of the Golgi apparatus fluctuates according to physiological demands or pathological stress conditions. For instance, in professional secretory cells such as goblet cells and secretory mucous cells of the Blunner’s gland, the size of the Golgi can be expanded [77,78]. In prolactin-secreting cells of the pituitary gland and acinar cells of mammary glands, the Golgi apparatus can be changed dynamically during lactation [79,80]. Under viral infection, the Golgi apparatus is increased or fragmented [81]. Besides these changes, there are reports indicating that when protein modification and secretion exceed the Golgi capacity there is a stress response (referred to as UPRgl) to increase the expression of glycosylation enzymes and vesicular transportation [82,83,84]. However, the Golgi apparatus is more complicated than the ER and the molecular mechanisms underlying UPRgl are not clear. There are no molecular sensors similar to the ER resident IRE1, PERK and ATF6 being identified in the Golgi. Nevertheless, a couple of pathways are found to be associated with the UPRgl (Figure 1 and Table 1). First is the TFE3 (a basic-helix-loop-helix type transcription factor) pathway [82,83]. TFE3 regulates transcriptional activation of genes collectively called UPRgl genes encoding Golgi resident protein 60 (GCP60), GM130, giantin, sialyltransferase 4A, fucosyltransferase 1, and syntaxin 3A, which generally encode Golgi structural proteins, glycosylation enzymes, or the vesicular transport components. Translocation of TFE3 into the nucleus depends on the status of its phosphorylation. Upon UPRgl, highly phosphorylated TFE3 in the cytoplasm is dephosphorylated and translocated into the nucleus activating the UPRgl genes. The second is the CREB3-ARF4 pathway, which involves both the ER and Golgi [85]. CREB3/Luman is a basic leucine zipper-containing transcription factor that resides in the ER membrane [86,87,88]. ARF4 is a member of the small GTPase family that regulates Golgi-to-ER vesicular transport via COP-I vesicles [89]. Cells treated with Brefeldin A that inhibits protein transport from the ER to the Golgi are under both ER and Golgi stress. Upon the ER stress, CREB3 is activated by proteolysis. Upon the Golgi stress, CREB3 upregulates the transcription of ARF4 in the nucleus, resulting in Golgi stress induced apoptosis. Another UPRgl pathway may be mediated by the HSP47. HSP47 is an ER chaperone. Expression of HSP47 is increased when glycosylation is inhibited. However, when the expression of HSP47 is suppressed by siRNAs, the Golgi apparatus fragmented leading to increased cell apoptosis [90]. This suggests that HSP47 may protect the cell from Golgi stress. Since the Golgi has a cis face and a trans face, it is likely that HSP47 and CREB3-ARF4 pathways regulate the function of the cis-Golgi network, whereas the TFE3 pathway regulates the trans Golgi network.

Alcohol has long been known to induce ultrastructural changes in the Golgi. In man and rat, chronic alcohol feeding with nutritionally adequate diets induced ultrastructural abnormalities of the intestinal epithelial cells [91]. Dilatation of the ER and cisternae of the Golgi apparatus were found in villus and crypt cells both in the jejunum and in the ileum. In lactating rats, chronic ethanol exposure resulted in a loss of the mammary cell polarization [92,93], which was specifically associated with an increased concentration of acetaldehyde, a reduction of the Golgi dictyosomal elements, and an altered casein maturation and secretion. In the supraoptic nucleus of the hypothalamus of ethanol-treated rats, prolonged alcohol ingestion leads to neuronal loss [94]. The surviving neurons display marked increase in volume, which was associated with an increased volume and surface area of the rough ER and Golgi apparatus as well as an altered transport and release of the neurosecretory materials [95,96], indicative of a presence of the Golgi stress. Similarly, in testes and accessory sex organs of man and animal models with chronic alcohol ingestion, a reduction in the glandular epithelium cell height was associated with disorganization of the Golgi complex [97]. In the alcohol stressed Golgi, abundant membrane-bound structures representing cytoplasmic material and accumulation of dense bodies were observed, which indicates negative effects of alcohol on the secretory process.

In addition to the above morphological changes, there is evidence for alcohol-induced functional and metabolic changes as a consequence of the Golgi stress. Chronic alcohol exposure is shown to affect the ER and Golgi apparatus involved in membrane traffic in neuronal dendrites [98]. Alcohol exposure reduced the volume and surface density of the rough ER, increased the levels of ER chaperone GRP78, and significantly diminished the proportion of neurons that show an extension of Golgi into dendrites and dendritic Golgi outposts, a structure present exclusively in apical dendrites. The alcohol effects on the Golgi outposts leads to negative repercussions for the development and maintenance of their polarized morphology and function. Several other studies linked alcoholic Golgi stress to metabolic changes. It was observed that ethanol altered glycosyl transferase activity in the hepatic Golgi apparatus [99]. Acute ethanol intoxication affects various steps of protein glycosylation at the level of rat Golgi apparatus [100]. Isolated hepatocytes pre-labelled with radioactive palmitate and glucosamine showed significant accumulation of lipid and carbohydrate radioactivity in microsomes and Golgi apparatus with a significant impairment of labelled glycolipoprotein secretion. Glycosyl transferase activities in the hepatocytes were significantly decreased in response to chronic alcohol. This disrupted secretion of glycolipoproteins could further trigger production of anticytoplasmic autoantibodies contributing to alcohol-induced liver disease. In fact, there is evidence showing the presence of high titers of anti-Golgi antibodies in human alcoholics with hepatocellular carcinoma (HCC) [101]. Moreover, the defects in the glycosylation in the stressed Golgi may also lead to molecular conformation changes of glycoproteins resulting in its structural instability and/or functional impairment. In rat brain, long-term ethanol treatment was shown to affect activities of brain sialylation and desialylation enzymes which led to a marked impairment in the glycosylation of clusterin [102]. Clusterin is an N-glycosylated sialoglycoprotein and its aggregation in brain cells has been suggested to contribute to caspase-3-independent brain injury [103]. In addition, alcohol exposure down regulates the sialyltransferase ST6Gal-I. ST6Gal-I mediates the glycosylation of proteins and lipids through adding the negatively charged sugar, sialic acid, in a α2-6 linkage to the termini of *N*-glycans to form functionally important glycoproteins and glycolipids in the Golgi compartment [104]. Long-term ethanol feeding in rats caused a marked decrease in ST6Gal-I activity as well as ST6GalI mRNA level in the liver. Clinical observations further showed that down-regulation of the ST6GalI gene and consequent impaired activity of ST6GalI was a major cause for the presence of sialoconjugates in the blood of long-term alcoholics. Intriguingly, the stability of the ST6GalI mRNA was demonstrated to be a mechanism for its impaired expression, which might be reminiscent of the regulated Ire1-dependent decay of mRNAs as a consequence of the ER stress [105]. Therefore, alcohol-induced Golgi stress and pathological consequences are evident despite that the molecular mechanisms underlying alcohol-induced UPRgl are obscure.

## 5. Alcohol and UPR in the Lysosomes

The lysosome is an organelle that contains acid hydrolase enzymes for breaking down molecular wastes and cellular debris [106]. This organelle involves three intracellular processes, namely phagocytosis, endocytosis and autophagy [107]. Extracellular materials are engulfed by phagocytosis and macromolecules are taken up by endocytosis. Proteins and other damaged cell organelles are recycled by autophagy. Autophagy plays an essential role in protein homeostasis, which involves formation of double membrane autophagosomes and fusion with lysosomes [108]. The autophagy-lysosome pathway together with the UPS forms two main pathways in turning over proteins of mammalian cells. The UPS including ERAD, preferentially degrades soluble and short-lived proteins whereas the autophagy-lysosome pathway degrades long-lived or aggregated proteins [108,109,110,111]. There is a molecular switch mechanism regulated by the Bcl-2e associated athanogen (BAG) proteins, which mediate whether proteins are delivered to the UPS or to the autophagy-lysosome [111,112]. The two pathways thus influence cellular protein homeostasis and are often related to the status of lysosomal functionality. Impaired ERAD under ER stress could increase protein load/cargo of lysosomes. Deficiency in a single lysosomal enzyme could prevent breakdown of target molecules and consequently lead to an accumulation of the un-degraded materials such as misfolded or aggregated proteins within the lysosomes, which often gives rise to severe clinical symptoms such as lysosomal storage diseases [114]. Conceptually, such stress would initially trigger the lysosome stress response (referred to as UPRly) to increase the capacity of the lysosome [108]. The kinase complex mTORC1 (mammalian target of rapamycin complex 1) may regulate UPRly and autophagy initiation (Figure 1 and Table 1) [115]. Under conditions with high levels of amino acids, mTORC1 is recruited to the lysosomal membrane by the v-ATPase-Regulator complex, and activated by the small GTPase Rheb. Activated mTORC phosphorylates S6K (ribosomal protein S6 kinase) and 4E-BP1 (eukaryotic initiation factor 4E-binding protein 1), which enhances translation and cell growth. Phosphorylation of ULK1 (unc-51 like autophagy activating kinase 1) and the transcription factor TFEB (transcription factor EB) [116,117] results in suppression of autophagy. These processes can be reversed upon starvation of amino acids resulting in expanded lysosomal compartments and activation of autophagy [118].

Early studies revealed that alcohol induced lysosomal abnormalities; in the hippocampal pyramidal cells of rats exposed to chronic alcohol, the volumetric density of lysosomes was increased significantly [119]. Prolonged alcohol inhibited enzymatic hydrolytic mechanisms in multi-vesicular bodies resulting in increased transitional forms of the vesicular bodies towards lysosomes. In the cerebellum and hippocampus of the rat medial prefrontal cortex, prolonged alcohol induced alterations of the neuronal lysosomal system in the pyramidal cells [120]. Transitional forms of lysosomes towards lipofuscin granules composed of lipid-containing residues of lysosomal digestion were more numerous among alcohol-fed animals. Besides the lysosomal abnormalities, alcohol also exerts effects on lysosomal enzyme levels and activities. In the pure pancreatic juice obtained by direct cannulation of the main pancreatic duct of chronic alcoholics, concentrations of lysosomal hydrolases such as trypsin and amylase were increased compared to the juice of normal healthy volunteers [121]. In animal models, alcohol caused hyperamylasemia, pancreatic edema and pancreatic histological changes, which were associated with a redistribution of lysosomal enzyme cathepsin B from the lysosomal fraction to the zymogen fraction in subcellular fractionation [122]. Such a co-localization of lysosomal hydrolases with digestive enzymes in the same subcellular compartment indicates one mechanistic cause for alcoholic pancreatitis. In addition, it is well known that chronic alcohol consumption can cause protein accumulation and hepatomegaly [123,124,125]. Alcohol-caused deficiency in lysosomal cathepsins by altering their biosynthesis and/or their trafficking into lysosomes has been shown to contribute to decreased hepatic protein degradation [124]. Moreover, there is direct/indirect molecular evidence indicative of involvement of the lysosomal stress response. Alterations of the UPRly components including 4E-BP1, S6K and eIF4E were associated with inhibited myocardial protein synthesis in heart disease, which represents an important etiology of mortality in chronic alcoholics [126]. Chronic ethanol administration decreased the abundance of eIF4G associated with eIF4E in the myocardium, increased the abundance of 4E-BP1 associated with eIF4E, and reduced the extent of S6K phosphorylation. In the hippocampus of alcoholics, expression of over six hundreds genes was altered by chronic consumption of alcohol [127]. Among them, pathways related to inflammation, hypoxia and stress were activated, and pathways that play roles in neurogenesis and myelination were decreased. BAG3 was suppressed in the alcoholics, indicating that the molecular switch between the ERAD and autophagy for protein degradation was compromised, which might contribute to alcohol-induced neurodegeneration.

## 6. Alcohol and Inter-Organellar Crosstalk

The ER, Golgi apparatus and lysosomes are major components of the endomembrane system of the cell, which plays critical roles in cell homeostasis. Although each of the membranous organelles forms a single functional unit and their membranes cannot fuse with each other directly, they communicate through vesicle transport or inter-organellar connections [128,129]. The inter-organellar connections also exist between membranes of the ER and the nuclei or mitochondria. These connections enable inter-organellar information exchange, pathway coordination, or crosstalk during the stress response, which leads to either recovery of cellular homeostasis or facilitation of disease development. For instance, the activation of ATF6 requires both the ER and Golgi apparatus. Upon ER stress, phosphorylated ATF6 is transported to the Golgi in a COP-II vesicle, and sequentially cleaved by S1P and S2P proteases, resulting in release of the cytoplasmic domain of ATF6 [130]. The activated ATF6 mediates the UPR survival response involving an arrest of general protein synthesis and selected synthesis of chaperone proteins needed for ER homeostasis [5,6]. CHOP was initially found to mediate ER stress caused cell death by alcohol [72]. CHOP is also increased upon accumulation of unfolded proteins in the mitochondrial matrix, which mediates the UPRmt [66,73]. The expression of the ER-resident HSP47 can be induced by the Golgi stress, which protects cells from Golgi stress-induced apoptosis [90]. In addition, it is known that acute or chronic alcohol inhibits the ER resident SERCA in human and mouse hepatocytes [55] and in neurons of mouse spinal cord and rat Purkinje [131]. Since there is a SERCA-mediated intricate relationship between the ER and mitochondria in intracellular calcium homeostasis [131], the alcoholic inhibition of SERCA could accommodate a massive transfer of calcium from the ER to mitochondria resulting in worsened ER stress-caused cell death [132].

In some alcoholic disease development, crosstalk may involve multiple components of protein homeostasis among organelles, in which molecular chaperones play a central role. For example, the HSP70 family plays a role as molecular chaperons. Chronic maternal alcohol consumption has been shown to increase HSP70 in the developing brain of rats, which is associated with a characteristic pattern of neuroanatomy and biochemical changes, a cluster of symptoms called Fetal Alcohol Syndrome (FAS) [133]. Since the chaperones mediate ER stress as well as autophagy [134,135], the FAS may result from impaired protein degradation involving both the ER and the lysosomes. In the brain cells, abnormal proteins can be removed either through autophagy or through the ERAD-proteasome pathway. The BAG proteins modulate the switch between autophagy and ERAD through competing for binding of the adapter proteins, p62 and NBR (neighbor of BRC1), which shuttle the proteins to autophagic vesicles, or p97, which shuttles the proteins to the proteasome [112,113,136,137]. Chronic alcohol intake affects both p62 and BAG3 [41,42,138,139], which could break the molecular switch and disrupt the balance between the ER and lysosomes for protein degradation. Downstream of the disruption, autophagy cannot intervene and prevent cell death by removing the accumulated proteins and aggregates, whereas the UPR may transition into apoptosis by up-regulation of CHOP and downstream GADD34 (growth arrest and DNA damage 34) [137,140,141,142], which leads to the development of fetal alcohol syndrome. Thus, inter-organellar crosstalk could better explain the pathogenesis of some complex diseases induced by alcohol.

## 7. Conclusions

The current theory regarding the proteomic stress response in the ER is that ER-specific sensor molecule(s) detect impaired protein homeostasis and initiate stress signaling from the ER to the nucleus, which activates transcription factor(s) to regulate expression of genes related to the ER function resulting in either restoration of homeostasis under mild conditions or cell death injuries under severe conditions. The alcohol-induced ER stress response has well been established and fits this theory. Because alcohol easily crosses cellular membranes and affects all compartments inside the cell, it is logical to ask whether any protein stress activated mechanisms similar to the ER exist in other cellular organelles. The answer appears positive despite the fact that the underlying mechanisms are quite diverse and different among organelles (Figure 1). Essentially, the Golgi may respond to alcohol-induced proteomic stress by increasing glycosylation enzyme expression and vesicular transport components to prevent damages upon accumulation of misfolded proteins. The alcoholic mitochondrial stress response may increase mitochondrial chaperone and protease expression to cope with unfolded proteins accumulated in the mitochondria. Alcohol impairs autophagy and triggers the lysosome stress response to increase expression of lysosome genes such as cathepsins to facilitate protein turnover. Multiple organelle protein stress responses and their relationship to alcohol-induced organ disorders are an evolving field of interest. However, there are many challenges. Potential initiation factors and sequence of events of the mitochondrial, Golgi and lysosomal stress responses upon alcohol exposure are unknown, and require systemic analyses. The detailed injury mechanisms downstream of the stress response in these organelles are lacking. In addition, the complex signaling network of inter-organellar crosstalk leading to additive or synergistic cell death injuries is not yet clear; its clarification may help to explain the complexity of alcoholic disorders. Future studies focused on molecular details to identify their pathophysiological roles, could lead to the identification of new drug targets and the development of therapeutic approaches for delaying or preventing alcohol-induced diseases.
